# Predictors of limb saving in diabetic foot ulcer

**DOI:** 10.12669/pjms.40.7.9182

**Published:** 2024-08

**Authors:** Nizamud Din, Shaista Kanwal, Azizul Hasan Aamir, Tahir Ghaffar

**Affiliations:** 1 Nizamud Din, FCPS Medicine. Department of Diabetes, Endocrinology and Metabolic Diseases, MTI Hayatabad Medical Complex, Peshawar, Pakistan; 2 Shaista Kanwal, FCPS Medicine, FCPS Endocrinology, MRCP. Department of Diabetes, Endocrinology and Metabolic Diseases, MTI Hayatabad Medical Complex, Peshawar, Pakistan; 3 Azizul Hasan Aamir, MRCP, FRCP (Edin), FACE. Department of Diabetes, Endocrinology and Metabolic Diseases, MTI Hayatabad Medical Complex, Peshawar, Pakistan; 4 Tahir Ghaffar, FCPS Medicine, FCPS Endocrinology, MRCP. Department of Diabetes, Endocrinology and Metabolic Diseases, MTI Hayatabad Medical Complex, Peshawar, Pakistan

**Keywords:** Diabetic foot ulcer, Predictors, Limb salvage

## Abstract

**Objectives:**

This study was aimed to determine the various factors which could serve as predictor of saving of lower limb from amputation in patients with diabetic foot ulcer (DFU).

**Method:**

This three-year retrospective study was conducted in the Diabetes and Endocrinology Unit of Hayatabad Medical complex Peshawar, Pakistan. Demographic, clinical, laboratory and radiological information of the diabetic patients with DFU admitted between January 2020 to December 2022 was retrieved from the hospital files. Information regarding initial and final decision regarding amputation and the outcome of the ulcer was also recorded.

**Results:**

A total of 502 patients of diabetes mellitus (DM) with DFU were included in the study, of whom there were 279 (55.6%) males and 223 (44.4%) females. The mean age of the study population, mean duration of DM and mean HbA1c were 55.2 ± 9.8 years, 13.7 ± 6.7 years and 11.2 ± 2.4 %, respectively. Patients who had an amputation of their lower limbs had an increased age (p= 0.034), raised total leucocyte count (TLC) (p= <0.001), higher HbA1c (p= 0.025), had osteomyelitis (p= <0.001), and had a higher-grade ulcer (p= <0.001). On binary logistic regression analysis, ulcer grade (OR=7.4, p= <0.001), osteomyelitis (OR=11.8, p= <0.001), and initial decision of no amputation at the time of admission (OR=33.6, p=<0.001) were independently associated with the lower limb salvage.

**Conclusion:**

DFU which were of grade I to II, had no evidence of osteomyelitis and for which an initial decision was of no amputation were more likely to be salvaged.

## INTRODUCTION

Patients with Diabetes Mellitus (DM) have a 25% risk of developing foot complications in their lifetime and a 30-fold greater risk of having lower extremity amputation (LEA) compared to those who have no DM.^1^ The major risk factor for a non-traumatic lower extremity amputation is a diabetic foot ulcer (DFU).^2^ In a Systematic review, the global prevalence of DFU was reported to be 6.3%,^3^ while in Pakistan, its prevalence is 16.8%.^4^ These diabetic foot complications are associated with an increased health expenditure, with an estimated US $8659 cost per patient per Anum. This emphasizes the importance of early diagnosis and management of these ulcers.^5^

Several risk factors like peripheral neuropathy, peripheral arterial disease (PAD), diabetic kidney disease (DKD), infection and poor glycemic control have been found to be associated with DFU. Detailed evaluation of these ulcers, appropriate management of the vascular disease, antibiotic therapy for infection and local debridement of the wound are the standard practice for the management of DFU, however none of these have been identified to be predictive of limb salvage.^1^ Despite the efforts being made to manage DFU conservatively, some patients still undergoes some sort of LEA,^6^ which along with increased morbidity and mortality also have an impact on the emotional wellbeing and financial status of the patient.^7^

Most of the studies have assessed the risk factors for the DFU and LEA, however, very few studies have examined the factors which are predictive of limb salvage in patients with DFU. This study aimed to determine the various factors which could serve as predictive of saving of lower limb from amputation in patients with DFU. The prediction of these outcomes, such as limb salvage and amputation, would be of great value in guiding management and focusing on interventions for limb saving.

## METHODS

This three years retrospective study was conducted in the Diabetes and Endocrinology Unit of Hayatabad Medical complex Peshawar, Pakistan. Information of the diabetic patients with DFU, admitted between January 2020 to December 2022was retrieved from the hospital files and their identity was kept anonymous and confidential.

### Inclusion & Exclusion Criteria:

Both Type-1 and Type-2 DM patients of either gender with age ≥18 years, admitted with DFU were included in the study. Patients with traumatic foot ulcer, wounds due to malignancy, burns or pressure sores were excluded.

### Ethical Approval:

it was obtained from ethical committee of the hospital (Approval no. 1034 dated 06/09/2022).

All the relevant information such as demographics (sex, age, and DM duration), clinical information (grade and type of ulcer, sensory and motor neuropathy, PAD, DKD, retinopathy, ischemic heart disease (IHD), initial and final decision regarding amputation and outcome), laboratory investigation (HbA1c, total leucocyte count and wound culture sensitivity) and radiological investigation (osteomyelitis) were recorded from files.

DFU were classified into five grades according to the Wagner classification.^8^Sensory neuropathy was diagnosed on the basis of the patients ability to locate seven or less sites out of ten sites by using 10 gm monofilament.^9^ Peripheral arterial disease (PAD) was assessed with ankle brachial index (ABI) with an index< 0.8 considered to have PAD.^10^ Diabetic nephropathy was assessed by measurement of urine albumin to creatine ratio (ACR) in the spot urine sample, with an ACR of > 30 mg/g diagnosed to have diabetic nephropathy.^11^ Diabetic retinopathy was assessed by fundus examination by direct ophthalmoscopy followed by digital retinal camera imaging for the finding’s confirmation.^12^ Ischemic heart disease (IHD) was assessed through electrocardiogram and echocardiography.^13^

The primary outcome was either an amputation (minor or major) or a salvage of the lower extremity. Minor amputation was defined by amputation of the phalanx or at the metatarsal level, whereas major amputation was defined by amputation of the below-knee or above-knee level.^14^. The presence of osteomyelitis was confirmed either by clinical findings (positive probe to bone test) or radiological features (presence of periosteal thickening, osteopenia, erosion of the cortex, and new bone formation).^15^

### Statistical Analysis:

Data analysis was accomplished through SPPSS version 20. Continuous data was represented as means and standard deviations, whereas frequencies and percentages were calculated for categorical variables. Association of various predictors with the lower limb salvage and amputation was accomplished by utilizing the chi-square test. Binary logistic regression was employed to establish certain cofactors which are associated with the salvage of the lower extremity. All the p values were two sided, considered statistically significant if it was <0.05.

## RESULTS

A total of 502 DM patients with DFU were included, of whom there were 279 (55.6%) males and 223 (44.4%) females. The mean age of the study population was 55.2 ± 9.8 years. The mean duration of DM, HbA1c and TLC were 13.7 ± 6.7 years, 11.2 ± 2.4 % and 15.2 ± 5.5/mm, respectively. At the time of admission, the initial decision regarding the amputation was that of no amputation in 243 (48.4%) patients, minor amputation in 161 patients (32.1%) and that of major amputation in 98 (19.5%) participants. On the other hand, the final decision regarding amputation status was that of no amputation in 215 patients (42.8%), minor amputation in 146 (29.1%) subjects and of major amputation in 141 (28.1%) patients. Regarding the outcome, 206 (41%) subjects had no amputation while 296 (59%) patients had amputation. One hundred and fifty-six (31.1%) patients had grade I and II ulcers whereas 346 (68.9%) patients had grade III to V ulcers.

Various factors were analyzed for their association with the lower limb amputation and the results are presented in Table-I. It is evident that patients who finally ended to have an amputation of their lower limbs were of increased age (p= 0.034), had a greater TLC (p= <0.001), an increased HbA1c (p= 0.025), had evidence of osteomyelitis (p= <0.001), had nephropathy (p= 0.03) and had a higher grade of the DFU (p= <0.001). Patients with an initial decision of amputation also had a statistically significant association with the final fate of amputation (p= <0.001)

**Table-I:** Association of various predictors with the lower limb amputation and salvage.

Study variable		Lower limb Amputation status		Total(n=502)	p value

		Salvage (n=206) (%age)	Amputation (n=296) (%age)		
Gender	Male	107 (51.9)	172 (58.1)	279 (55.6)	0.17
	Female	99 (48.1)	124 (41.9)	223 (44.4)	
Age (years)	18-25	5 (2.4)	2 (0.6)	7 (1.4)	0.034
	26-40	14 (6.7)	22 (7.4)	36 (7.2)	
	41-60	149 (72.3)	189 (63.8)	338 (67.3)	
	>60	38 (18.4)	83 (28.04)	121 (24.1)	
Duration of DM (years)	1-10	86 (41.7)	103 (34.8)	189 (37.6)	0.21
	11-20	96 (46.6)	147 (49.7)	243 (48.4)	
	>20	24 (11.6)	46 (15.5)	70 (13.9)	
HbA1c (%)	6.5-8	25 (12.1)	16 (5.4)	41 (81.7)	0.025
	8.1-10	72 (34.9)	113 (38.2)	185 (36.8)	
	>10	109 (52.9)	167 (56.4)	276 (54.9)	
Total leucocyte count	4000-11000	66 (32.03)	51 (17.2)	117 (23.3)	<0.0001
	11100-15000	72 (34.9)	101 (34.1)	173 (34.5)	
	15100-30000	66 (32.03)	137 (46.3)	203 (68.6)	
	>30000	2 (0.9)	7 (2.4)	9 (1.8)	
Grade of Ulcer	Grade I	8 (3.9)	2 (0.7)	10 (1.9)	<0.0001
	Grade II	134 (65.04)	12 (4.1)	146 (29.1)	
	Grade III	58 (28.1)	82 (27.7)	140 (27.9)	
	Grade IV	6 (2.9)	168 (56.8)	174 (58.8)	
	Grade V	0 (0)	32 (10.8)	32 (6.4)	
Initial decision regarding amputation	No Amputation	198 (96.1)	45 (15.2)	243 (48.4)	<0.0001
	Minor amputation	6 (2.9)	155 (52.4)	161 (32.1)	
	Major amputation	2 (0.9)	96 (32.4)	98 (19.5)	
Sensory neuropathy	No	54 (26.2)	65 (21.9)	119 (23.7)	0.27
	Yes	152 (73.8)	231 (78.1)	383 (76.3)	
Motor neuropathy	No	123 (59.7)	152 (51.3)	275 (54.8)	0.06
	Yes	83 (40.3)	144 (48.6)	227 (45.2)	
IHD	No	129 (62.6)	181 (61.1)	310 (61.8)	0.7
	Yes	77 (37.4)	115 (38.9)	192 (38.2)	
PAD	No	45 (21.8)	78 (26.4)	123 (24.5)	0.25
	Yes	161 (78.2)	218 (73.6)	379 (75.5)	
CVD	No	184 (89.3)	263 (88.9)	447 (89.1)	0.9
	Yes	22 (10.7)	33 (11.1)	55 (10.9)	
Retinopathy	No	49 (23.8)	51 (17.2)	100 (19.9)	0.07
	Yes	157 (76.2)	245 (82.8)	402 (80.1)	
Nephropathy	No	68 (33.1)	72 (24.3)	140 (27.9)	0.03
	Yes	138 (66.9)	224 (75.7)	362 (72.1)	
Osteomyelitis	No	181 (87.9)	37 (12.5)	218 (43.4)	<0.0001
	Yes	25 (12.1)	259 (87.5)	284 (56.6)	
Wound Culture	No growth	43 (20.9)	39 (13.2)	81 (16.1)	0.02
	Yes	163 (79.1)	257 (86.8)	421 (83.9)	

**Table-II:** Logistic Regression Analysis of different predictors of limb salvage.

Variable		Odds Ratio	Confidence Interval	p value
Gender	Female	1.0		0.97
	Male	0.99	0.45-2.2	
Age (years)	>60 years	1.0		
	41-60	1.98	0.81-4.9	0.14
	26-40	1.2	0.22-6.64	0.8
	18-25	4.6	0.008-2663.7	0.6
Duration (years)	>20	1.0		
	10-20	1.27	0.35-4.6	0.7
	1-10	1.4	0.4-5.4	0.6
HbA1c (%)	>10	1.0		
	8.1-10	1.6	0.7-3.7	0.3
	6.5-8	3.9	0.7-22.7	0.13
Total Leucocyte Count (TLC)	>30,000	1.0		
	15100-30000	3.4	0.21-54.7	0.4
	11100-15000	4.6	0.3-77.4	0.3
	4000-11000	8.3	0.5-151.4	0.15
Grade of Ulcer	Grade III to V	1.0		
	Grade I and II	7.4	2.8-19.6	<0.0001
Culture	Bacterial Growth	1.0		
	No Bacterial growth	1.2	0.4-3.6	0.8
Nephropathy	Yes	1.0		
	No	1.5	0.6-3.8	0.4
Retinopathy	Yes	1.0		
	No	0.7	0.3-2.0	0.5
Sensory Neuropathy	Yes	1.0		
	No	0.8	0.3-2.1	0.6
Peripheral arterial Disease (PAD)	Yes	1.0		
	No	1.8	0.7-4.7	0.24
Osteomyelitis	Yes	1.0		<0.0001
	No	11.8	5.1-27.5	
Initial Decision regarding amputation	Major Amputation	1.0		
	Minor Amputation	0.96	0.2-5.3	0.96
	No Amputation	33.6	7.04-160.7	<0.0001

On binary logistic regression analysis, age, HbA1c, TLC, nephropathy and wound culture were no longer independent factors for limb amputation. It was evident that grade of ulcer, osteomyelitis, and initial decision regarding amputation at the time of admission were independently associated with the salvage of limb. It was observed that compared to HbA1c of >10 %, those who had an HbA1c of 6.5-8 % were more likely to have salvage of the lower limb (OR= 1.3, p= 0.3). Compared to grade III to V ulcers, those who had grade I to II ulcers were more likely to have salvage of the lower limb (OR=7.4, p= <0.001). In contrast to patients who had evidence of osteomyelitis, those who had no osteomyelitis were 11.8 times (p=<0.001) more likely to have salvage of their limbs. Those who had an initial decision of no amputation at the time of diagnosis were 33.6 times (p= <0.001) more likely to have limb salvage.

## DISCUSSION

This study demonstrated that various factors were associated with salvage of lower limbs in patients with DFU. It was evident that advanced age (p=0.034), increased TLC (p= <0.0001) and DKD (p=0.03) were associated with lower extremity amputation (LEA), however none of them was found to be independent factor for limb salvage in the logistic regression model. A study in Pakistan revealed 26.6% amputation rate in more infected ulcers.^16^ Study by Kurniawati et al. revealed that patients with DFU whose age was >50 years were more likely to undergo LEA compared to those whose age was <50 years.^17^

Study by Choi et al. revealed that higher TLC is associated with an increased risk of LEA and those with CKD had failure of limb salvage.^18^ Patients with better glycemic control were less likely to undergo LEA (p=0.025), however it was not determined to be an independent factor for limb salvage. A similar study in Pakistan also did not find an association between LEA and glycemic control.^19^ Study by Kim et al demonstrated that patients who had an HbA1c>9% were more likely to have an amputation compared to those whose HbA1c was < 9 % (42.9% versus 39.7%). However, this difference was not statistically significant.^8^

Factors like duration of DM, neuropathy, retinopathy, PAD, IHD and CVD were not associated with limb salvage, with none of them found to be an independent factor for limb salvage in the logistic regression model. Study by Aydin et al revealed that these factors are not associated with LEA.^20^ Though, a previous study conducted in Pakistan reported that factors like neuropathy, retinopathy and poor compliance with the medication was associated with an increased risk of DFU. However, they did not evaluate the effect of these factors on the risk of amputation in these ulcers.^21^ Our study revealed an association of osteomyelitis with LEA (p=<0.0001), but it was not an independent factor for limb salvage in the logistic regression analysis. Ulcers with no evidence of osteomyelitis were 11.8 times more likely to be saved from amputation (p=<0.0001). In a study by Wukich et al., it was found that patients who had osteomyelitis were 5.6 times more likely to have an amputation (p=<0.0001).^22^

The initial decision [no amputation= 243 (48.4%), minor amputation = 161 (32.1%) and major amputation = 98 (19.5%)] had a significant impact on the outcome [no amputation= 206 (41%) and amputation = 296 (59%)]. A study in Indonesia revealed that 36.3% subjects with DFU had a LEA.^23^ A study by Saleem et al found that 68% of the DFU healed completely, while 27.7% had an amputation.^24^ The difference in the outcome between our study and study in Indonesia could be attributed to the presence of osteomyelitis (51.6% versus 34.8%).^23^ Grade I to II ulcers were 7.4 times likely to have a healing and salvage of their lower limbs compared to those who had a higher grade ulcer. A study in Pakistan by Riaz et al. demonstrated a lower amputation rate in low grade ulcers.^4^ Likewise, Spanos K et al. also revealed that ulcer healing and limb salvage was more reported in lower grade ulcers.^25^ A study by Wang et al. revealed similar findings to our study. In their study, they reported that an increased size of ulcer (p=0.001), higher Wagner classification grades (p=0.002), and osteomyelitis (p=0.0001), were independent risk factors of LEA in patients with diabetic foot ulcers.^26^ Likewise, ulcers which showed no bacterial growth on culture were 1.2 times more likely to be salvaged. A study by Matta-Gutierrez G et al revealed that the evidence of bacterial growth was associated with the lower extremity ampuatation.^27^

### Limitations:

This study analyzed the various factors to be predictive of limb salvage in patients with DFU in detail and provided evidence of the predictive factors in our population. It had enough sample size; thus, results can be considered reliable. It had a few limitations. Firstly, this study was a single center study, thus the findings cannot be generalized. Secondly, the ulcers which were managed conservatively were not monitored to ascertain their ultimate outcome.

## CONCLUSION

Our study demonstrated that DFU which were of grade I to II, had no evidence of osteomyelitis and for which an initial decision was of no amputation were more likely to be saved from a lower extremity amputation. Other factors like age, duration of DM, glycemic control, bacterial growth, infection and micro and macrovascular complications were not associated with the lower limb salvage.

### Author’s Contribution:

**ND:** Conceived, designed, did literature review, performed statistical analysis & drafted the manuscript.

**SK:** Participated in analysis, literature review and interpretation of data, and helped in drafting the manuscript.

**AHA:** Conceived, designed, and critically revised the manuscript.

**TG:** Participated in data collection, did literature review and interpretation of data. All authors provided final approval for publication of the manuscript and are responsible for the integrity of the study.

## References

[1] Yakoot M, Abdelatif M, Helmy S Efficacy of a new local limb salvage treatment for limb-threatening diabetic foot wounds-a randomized controlled study. Diabetes, Metabolic Syndrome and Obesity:Targets Ther.

[2] Lavery LA, Armstrong DG, Wunderlich RP, Mohler MJ, Wendel CS, Lipsky BA (2006). Risk factors for foot infections in individuals with diabetes. Diabetes care.

[3] Zhang P, Lu J, Jing Y, Tang S, Zhu D, Bi Y (2017). Global epidemiology of diabetic foot ulceration:a systematic review and meta-analysis. Ann Med.

[4] Riaz M, Miyan Z, Waris N, Zaidi SI, Tahir B, Fawwad A (2019). Impact of multidisciplinary foot care team on outcome of diabetic foot ulcer in term of lower extremity amputation at a tertiary care unit in Karachi, Pakistan. Int Wound J.

[5] Raghav A, Khan ZA, Labala RK, Ahmad J, Noor S, Mishra BK (2018). Financial burden of diabetic foot ulcers to world:a progressive topic to discuss always. Ther Adv Endocrinol Metab.

[6] Nather A, Siok Bee C, Keng Lin W, Xin-Bei Valerie C, Liang S, Tambyah PA (2010). Value of team approach combined with clinical pathway for diabetic foot problems:a clinical evaluation. Diabetic foot &ankle.

[7] Cho MK, Kwon SB, Kim CH, Lee YJ, Nam HS, Lee SH (2014). Overexpression of KAI1 protein in diabetic skin tissues. Arch Plastic Surg.

[8] Kim SY, Kim TH, Choi JY, Kwon YJ, Choi DH, Kim KC (2018). Predictors for amputation in patients with diabetic foot wound. Vasc Specialist Int.

[9] Fernando ME, Crowther RG, Pappas E, Lazzarini PA, Cunningham M, Sangla KS (2014). Plantar pressure in diabetic peripheral neuropathy patients with active foot ulceration, previous ulceration and no history of ulceration:a meta-analysis of observational studies. PloS one.

[10] Pasqualini L, Schillaci G, Pirro M, Vaudo G, Leli C, Colella R (2012). Prognostic value of low and high ankle-brachial index in hospitalized medical patients. Eur J Intern Med.

[11] Nah EH, Cho S, Kim S, Cho HI (2017). Comparison of urine albumin-to-creatinine ratio (ACR) between ACR strip test and quantitative test in prediabetes and diabetes. Ann Lab Med.

[12] Malik SE, Ghaffar T, Ullah Z, Kanwal S, Noor A, Aamir Azizul H (2021). Frequency of unrecognised diabetic retinopathy and nephropathy in patients presenting with diabetic foot ulcers. J Postgrad Med Inst.

[13] Baggiano A, Italiano G, Guglielmo M, Fusini L, Guaricci AI, Maragna R (2022). Changing Paradigms in the Diagnosis of Ischemic Heart Disease by Multimodality Imaging. J Clin Med.

[14] Perng CK, Chou HY, Chiu YJ (2021). Identifying major predictors of lower-extremity amputation in patients with diabetic foot ulcers. J Chinese Med Assoc.

[15] Malhotra R, Chan CS, Nather A (2014). Osteomyelitis in the diabetic foot. Diabetic Foot Ankle.

[16] Yasin M, Zafar S, Rahman H, Khan TA, Nazir S, Shah S (2018). Baseline characteristics of infected foot ulcers in patients with diabetes at a tertiary care hospital in Pakistan. J Wound Care.

[17] Kurniawati A, Ismiarto YD, Hsu IL (2019). Prognostic factors for lower extremity amputation in diabetic foot ulcer patients. J Acute Med.

[18] Choi MS, Jeon SB, Lee JH (2014). Predictive factors for successful limb salvage surgery in diabetic foot patients. BMC Surg.

[19] Ali SM, Basit A, Fawwad A, Ahmedani MY, Miyan Z, Malik RA (2008). Presentation and outcome of diabetic foot at a tertiary care unit. Pak J Med Sci.

[20] Aydin K, Isildak M, Karakaya J, Gürlek A (2010). Change in amputation predictors in diabetic foot disease:effect of multidisciplinary approach. Endocrine.

[21] Khan MIH, Azhar U, Zubair F, Khan ZA (2018). Can we link foot ulcer with risk factors in diabetics?A study in a tertiary care hospital. Pak J Med Sci.

[22] Wukich DK, Hobizal KB, Sambenedetto TL, Kirby K, Rosario BL (2016). Outcomes of osteomyelitis in patients hospitalized with diabetic foot infections. Foot Ankle Int.

[23] Pemayun TG, Naibaho RM (2017). Clinical profile and outcome of diabetic foot ulcer, a view from tertiary care hospital in Semarang, Indonesia. Diabetic foot ankle.

[24] Saleem S, Hayat N, Ahmad I, Ahmad T, Rehan A (2017). Risk factors associated with poor outcome in diabetic foot ulcer patients. Turk J Med Sci.

[25] Spanos K, Saleptsis V, Athanasoulas A, Karathanos C, Bargiota A, Chan P (2017). Factors associated with ulcer healing and quality of life in patients with diabetic foot ulcer. Angiology.

[26] Wang L, Li Q, Chen X, Wang Z (2022). Clinical characteristics and risk factors of lower extremity amputation in patients with diabetic foot. Pak J Med Sci.

[27] Matta-Gutierrez G, Garcia-Morales E, Garcia-Alvarez Y, Álvaro-Afonso FJ, Molines-Barroso RJ, Lazaro-Martinez JL (2021). The influence of multidrug-resistant bacteria on clinical outcomes of diabetic foot ulcers:a systematic review. J ClinMed.

